# Grain Quality Characterization before and after Processing

**DOI:** 10.3390/foods12203728

**Published:** 2023-10-11

**Authors:** Yanhui Li, Yue Kong, Zenan Wu, Fengying Xie

**Affiliations:** College of Food Science, Northeast Agricultural University, Harbin 150030, China; liyanhui3511@163.com (Y.L.); kyyy0531@163.com (Y.K.); 18800429312@163.com (Z.W.)

## 1. Introduction

Grains are an important part of a healthy diet, and provide most of the daily calories and nutrients. According to the USDA Dietary Guidelines 2020–2025, a healthy adult should eat an average of six one-ounce portions of grain a day, half of which should be whole grain. The main grains grown worldwide are wheat, maize, and rice, as well as rye, barley, buckwheat, oats, etc. The OECD-FAO Agricultural Outlook 2023–2032 shows the basic facts of the world grain situation: that global consumer demand for cereal production growth is expected to slow over the next decade, partly because the per capita consumption of most cereals is approaching saturation in many countries. It is projected that by 2032, 41% of all cereal production will be used for food consumption, 37% for feed production, and the remainder will be consumed through the production of biofuels and other industrial uses [1]. Grain is an important component of food production, and its processing is an important link in reducing losses and improving product quality. Empowering science and technology can help in the scientific and technological innovation of health and nutritional foods, and lead to a new trend of grain diets.

## 2. Grain Processing

Grain undergoes primary processing for the removal of impurities from the raw grain, the regulation of moisture, and for hulling, peeling, or grinding. It is then further processed into finished granular grains and their products, which must meet different quality standards. Grain processing is accomplished by applying heat, pressure, mechanical action, or various combinations thereof. Among them, mechanical treatments such as roller and hammer mills used in primary processing can efficiently separate the bran from the endosperm. Heat treatments (roasting, bursting, micronizing, autoclaving, steam flaking, steam pelletizing, swelling, extruding, and roasting) can ripen the grain, reduce populations of spoilage microorganisms, and deactivate unwanted enzymes and anti-nutritional factors, thereby extending the shelf life of stored products. In addition, some biological treatment processing techniques (germination, fermentation, and enzymolysis) are also involved in grain processing, and can increase the content of some bioactive compounds and even synthesize new compounds [2]. In food manufacturing, the processing properties of grain are significant. For example, during milling, soundness is the most important factor to consider when it comes to the quality of grain. The need to remove hulls or to improve quality during processing thus determines the edible yield and economic value of the grain. However, for consumers, properties such as color, flavor, and nutrition are most important. The seed coat and germ of the grain are very rich in nutrients, and are a large source of good macronutrients, micronutrients (B vitamins, minerals, etc.), dietary fiber, and many bioactive components. During refining processes, such as for rice and white flour, 60–90% of these nutrients and active substances are lost. In addition, some grains are mechanically damaged, and the damaged grains absorb more water, which affects product quality characteristics such as taste and storage needs. Therefore, there is a need to study how processing technologies can change the quality characteristics of grains and how these processing technologies can be effectively used or improved to maintain or improve the quality of the grains.

## 3. Grain Quality Characterization

Grain quality characterization is a process of evaluating the quality of grains based on extrinsic and intrinsic factors. During primary processing, external factors such as age, broken grains, immature grains, foreign matter, infected grains, etc., and internal factors such as color, size, shape, and stacking density need to be taken into account. However, in further processing, quality characteristics such as the taste, odor, texture, and nutrition of the product also need to be assessed more closely. For example, during rice processing, temperature affects the browning and milling rate of rice and reduces the head rice rate, while in terms of appearance quality, it affects the chalkiness [3]. Moreover, high pressure treatments partially destroy the endosperm cells in the rice grain, leading to the release of allergenic proteins, making it possible not only to reduce the allergens in the grain, but also to improve the quality of the cooked rice [4]. Therefore, the quality of grains can be affected by several factors such as growing practices, processing, and storage management.

In addition, for the dominant grains such as wheat, maize, and rice, each major exporting country (or group of countries, e.g., Europe) has developed standards for grade and class. Color is an important primary factor in the characterization, grading, trading, and processing of grains, and is a common criterion used in the wheat trade. Size and shape are important factors affecting the quality and grading of grains and are commonly used in rice grading. The major components of grains may vary considerably due to different processing methods, which play an important role in the grading and marketing of grains and their further processed products. Therefore, the quality characteristics of processed grains have a key impact on their export trade, marketing, and high-value further processing, and the increasing demand for whole grain foods is progressively requiring quality characteristics in processed grains.

However, the mechanisms for the formation of grain quality and the lack of quality control are significantly restricting the grain industry’s development. This Special Issue aims to publish quality articles on grain quality characterization during processing, and is dedicated to changes in characterization and structure before and after processing, exploring the regulation mechanisms. Laboratory and pilot-scale quality characterization studies are useful to evaluate the potential performance of grains in processing and to measure the quality of the end products.

## Data Availability

Not applicable.
